# Retention in physically demanding jobs of individuals with low back pain: study protocol for a randomised controlled trial

**DOI:** 10.1186/s13063-015-0684-3

**Published:** 2015-04-16

**Authors:** Bjarke Brandt Hansen, Lilli Kirkeskov, Robin Christensen, Luise Mølenberg Begtrup, Ellen Bøtker Pedersen, Jakob Falk Teilya, Mikael Boesen, Gilles Ludger Fournier, Henning Bliddal, Ann Isabel Kryger

**Affiliations:** Department of Rheumatology, The Parker Institute, Copenhagen University Hospital, Bispebjerg and Frederiksberg Hospital, Nordre Fasanvej 57, 2000 Frederiksberg Copenhagen, Denmark; Department of Occupational and Environmental Medicine, Copenhagen University Hospital, Bispebjerg and Frederiksberg, Bispebjerg Bakke 23, DK-2400 Copenhagen, NV Denmark; Department of Radiology, Copenhagen University Hospital, Bispebjerg and Frederiksberg, Nordre Fasanvej 57, 2000 Frederiksberg Copenhagen, Denmark; Department of Rheumatology, Copenhagen University Hospital, Bispebjerg and Frederiksberg, Nordre Fasanvej 57, 2000 Frederiksberg Copenhagen, Denmark

**Keywords:** Low back pain, Sick leave, Workplace intervention, MRI, Weight-bearing, Occupational medicine, Physically demanding jobs, G-MRI

## Abstract

**Background:**

Low back pain is prevalent and is a frequent cause of disability and sick leave among working adults. Individuals with low back pain often consult general practice or other health care providers which often results in a unilateral intervention focussed on their symptoms. Employment is associated with physical and mental well-being, so, patients may benefit from an early additional occupational medicine intervention. For individuals with physically demanding jobs it can be especially challenging to retain their jobs. The aim of the ‘GoBack trial' is to develop and evaluate the efficacy and feasibility of an occupational medicine intervention for individuals with low back pain in physically demanding jobs.

**Methods/design:**

We will conduct a randomised controlled trial enrolling 300 participants with difficulty in maintaining physically demanding jobs due to low back pain for a current period of 2 to 4 weeks. Participants will be randomised and stratified according to their age and gender before being allocated in a 1:1 ratio to either control or additional occupational medicine intervention. Both groups will receive conventional treatment for their low back pain during the study. All participants will be thoroughly assessed for causes of low back pain and potential prognostic factors by questionnaires, clinical specialist assessments and magnetic resonance imaging (MRI) scans of the lumbar spine. Primary outcome is the accumulated duration of self-assessed sick leave (in days) due to low back pain during 6 months from baseline. Secondary outcomes include general self-rated back pain, disability and screening for potential prognostic factors: fear avoidance behaviour, disability, health status and degenerative MRI findings. For tertiary purposes selected outcomes will also be assessed after 1 and 2 years from baseline.

**Discussion:**

Many guidelines exist for the management of low back pain, but they provide limited guidance on occupational aspects. The findings from this randomised trial will provide high-quality evidence for the efficacy and feasibility of an occupational medicine intervention model for individuals with low back pain in physically demanding jobs.

**Trial registration:**

This trial was registered with ClinicalTrials.gov (identifier: NCT02015572) on 29 November 2013.

## Background

Low back pain (LBP) is a recognised public health problem, and the lifetime prevalence may be up to 80% in industrialised countries [1]. LBP is a common reason for doctor visits and is a significant cause of sick leave and disability pension with huge personal and socio-economic consequences [2]. Despite increased attempts of prevention, the incidence curves of LBP have still not been altered. The explanation is supposed to be that LBP and the resulting function deterioration are a complex combination of physiological, psychological, cultural and social factors. It is therefore recommended to focus on tertiary prevention in patients who have already developed LBP [3]. Medical treatment may reduce the physical and mental discomfort, while it has not been able to improve the possibilities for return to work [4]. Further, it has been demonstrated that over 60% of LBP patients from general practice may experience more than three episodes of pain after their first episode [5]. Twin studies indicate that disc degeneration and LBP correlate more closely with genetic heritability than with physical load [6,7]. Despite a large heterogeneity in LBP conditions, the overall picture is clearly that LBP is not a self-limiting condition [5,8,9]; therefore, return to work or work retention seems to be a good outcome measurement for LBP interventions.

Even with the multitude of different treatments offered to patients with LBP, there is no treatment that has been proven to be highly effective. In recent years focus has therefore been on cognitive behavioural therapy and the attachment to the labour market [10,11]. Occupational attachment is associated with physical and mental well-being [12], and there seems to be negative association between long-term sick leave and later labour market attachment [13]. Although there is little knowledge about prognostic factors, it seems that job satisfaction and opportunity to work adaptation are important factors for work rehabilitation [14]. Furthermore, early intervention including contact with the individual who is ill with LBP, workplace visits and effective cooperation between the individual, the employer and the clinical team may prevent permanent exclusion from the labour market [15]. From workplace and municipal intervention programs we know that occupational medicine intervention has an effect on work rehabilitation, albeit a small one [16]. The literature in this field is confusing due to considerable variations of the definitions of both LBP as well as recovery [17,18].

Fear avoidance behaviour has been shown to be an essential element of the disabling pathway in chronic LBP, and biopsychosocial models have been an established part of most interventions [19,20], but there is limited knowledge about the effect on occupational rehabilitation. Physical exercise alone has only limited influence on the work ability of LBP patients, whereas supervised training and a behavioural therapeutic approach seem to be much more effective in reducing sick leave [21]. Therefore, intervention for individuals on sick leave with physically demanding jobs should be based on cognitive behaviour treatment addressing fear avoidance behaviour towards the workplace, and job modifications in combination with supervised physical exercise.

The objective of this study is to evaluate whether an additional occupational medicine intervention with focus on early workplace oriented counselling and workplace intervention can retain individuals with physically demanding jobs and LBP in employment. The secondary aims are to identify prognostic factors for success of this occupational medicine intervention using the baseline outcomes: pain level, health status, fear avoidance behaviours, job satisfaction, work ability, and degenerative magnetic resonance imaging (MRI) findings. Among these variables, we aim to identify individuals who already have a good prognosis and therefore have no need for this larger scale intervention. Later, a cost-benefit analysis of the intervention will be performed.

## Methods/design

**Figure 1:**
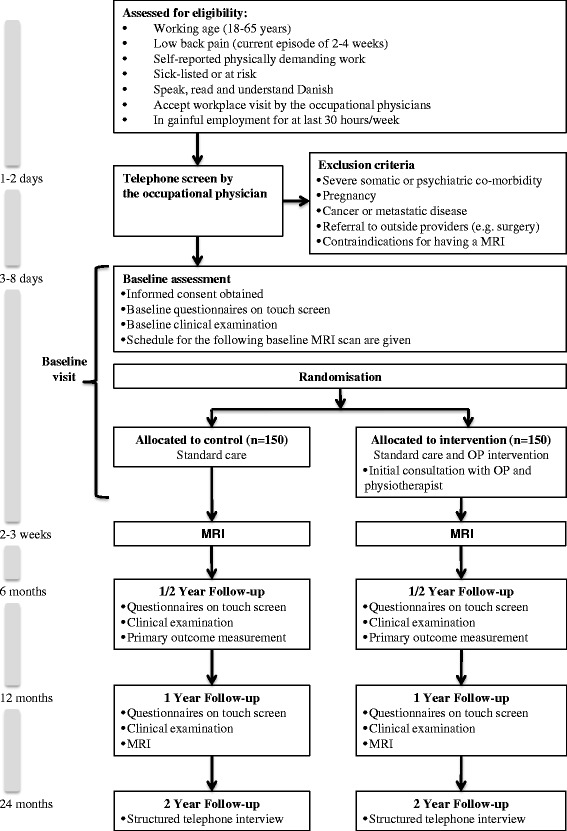
Participants flow through the study.

As illustrated in Figure 1, this is a randomised controlled trial with 1:1 allocation. It is conducted in accordance with the SPIRIT (Standard Protocol Items: Recommendations for Interventional Trials) 2013 guidelines [22]. The study is designed to investigate if an additional occupational medicine intervention can retain individuals with physically demanding jobs and LBP in gainful employment over a 6-month period. Eligible participants will be randomised after baseline measurements to one of two parallel groups: 1) control, 2) additional occupational medicine intervention. Data are collected at each stage of the trial, at initial telephone screening, allocation, occupational sessions and through all follow-up visits, and are reported according to CONSORT (Consolidated Standards of Reporting Trials) statements [23]. The study is approved by the Local Research Ethics Committee, Region H, Denmark (H-3-2013-161) 20 November 2013.

### Inclusion criteria

Eligible participants are persons between 18 and 65 years of age with a current episode of 2 to 4 weeks of LBP with a self-reported physically demanding job, who independently of sick leave status, express concerns about the ability to maintain their current job. They must be able to speak, read and understand Danish in such a way that they can give informed consent for participating. The potential participants have to be in employment for at last 30 hours/week and must accept workplace visits by an occupational physician (OP) or physiotherapist. According to Robreok *et al*. [24], physically demanding work is defined by a self-reported patient statement using a single question: *Would you strongly agree, agree, disagree or strongly disagree that your work is physically demanding?* Those individuals who strongly agree or agree are considered to have physically demanding work. LBP is defined as pain located in the lower back with or without radiating symptoms and must fulfil International Classification of Diseases, 10th Revision (ICD-10) back pain codes: DM43.1; DM47; DM48.0; DM54.3-54.9; DM 51.1; DM51.2; DM51.3; DM 51.9; DM53.8; DM53.9. [http://apps.who.int/classifications/icd10/browse/2010/en].

### Exclusion criteria

The exclusion criteria are pregnancy, severe somatic or psychiatric disease, cancer or metastatic disease, severe co-morbidity, treatment or referral to outside providers (for example, surgery) or contraindications for having a conventional MRI.

### Study settings and recruitment

Participants are recruited in the capital region of Copenhagen (Denmark) and the surrounding counties (Region H) with approximately 1.7 million residents. Physicians, health providers and general practitioners (GPs) will continuously be informed of the possibilities of referral to the project. The referrer may suggest participation to any patient with a current episode of 2 to 4 weeks of LBP and self-reported physically demanding work. Potential eligible patients will be referred to the Department of Occupational and Environmental Medicine, Copenhagen University Hospital, Bispebjerg and Frederiksberg, which will contact the candidate by telephone and screen for criteria of inclusion and exclusion. If needed, recruitment via advertisement can be an option. Individuals who fulfil the inclusion criteria are forwarded written information of the study and are scheduled within one week for the baseline visit at The Parker Institute, Department of Rheumatology, Copenhagen University Hospital, Bispebjerg and Frederiksberg.

Qualified physicians from the Parker Institute, Department of Rheumatology, will conduct all baseline and follow-up health assessments. They are not allowed to treat the participants or take action in the occupational medicine intervention but will take action if the physical examination reveals conditions which need further clinical intervention. Outcomes will be recorded in dedicated databases at baseline, half-a-year (primary endpoint), and at the one-year follow-up at The Parker Institute, Department of Rheumatology. The GPs are continually informed of the allocation and occupational medicine intervention plan. In addition, imaging data and subsequent interpretation are automatically stored on dedicated imaging servers within the capital region of Denmark (Region H) for 10 years as part of normal clinical practice. The 2-year follow-up is a structured interview conducted by independent non-treating personnel from the occupational department.

### Study timeline

The primary health provider (for example, the GP) can provide a letter of introduction, contact information and informed consent. Both the primary health provider and participants can find these documents and additional information on the trial’s dedicated website www.goback.dk (in Danish). The screening telephone interview by the occupational department’s staff takes about 10 to 15 minutes, and eligible participants are mailed the baseline visit schedule and the manual Guidance for Participants in Clinical Studies.

The enrolled participants meet at The Parker Institute, Department of Rheumatology, for baseline visit within a week after the screening interview. The institute’s staff reviews the informed consent document with the participant before signing. The participants complete the baseline questionnaires on a validated touch screen [25] with information including demographic and personal data, general health information, history of work-related factors, sickness absence, LBP pain score, fear avoidance and back-specific disability. A qualified physician then performs a clinical examination and reviews relevant health-related answers, noting if further information or examinations are necessary or there is a need for further referral. The physician completes the hospital’s web-based (OPUS) clinical report that includes systematic questions on key eligibility criteria, clinical findings, red flag conditions and a LBP-focussed case summary. This report is sent to the participant’s GP. When questionnaires and clinical examinations have been obtained, the randomisation takes place and the group allocations are revealed to the participants. The participants in the intervention group will have their first occupational medicine intervention session the same day.

Due to logistics, the participants will be scanned at baseline as a post-allocation examination within 2 weeks. As part of the one-year follow-up all participants will be rescanned in the same setting and by the same MRI protocol. The MRI scans take place at the Department of Radiology, Frederiksberg Hospital, and a 0.25-T tilting MRI unit (G-Scanner, ESAOTE, Genova, Italy) is used. The MRI scan includes conventional sagittal T2-weighted (T2w) and T1-weighted (T1w) turbo –spin echo (TSE) sequence and an axial and coronal TSE-T2w sequence covering all lumbar spine segments. In addition, prior to the conventional scanning, all participants will be scanned in the standing weight-bearing position, including a sagittal TSE-T2w sequence and axial and coronal TSE-T2w sequences. Due to the small dimension of the scanner and risk of orthostatic syncope, it may be necessary to convert to conventional supine high-field MRI. This assisting MRI scanning is evaluated for red flag conditions and potential exclusion criteria within 2 weeks, while the scientific semi-quantitative evaluation is performed in detail later. Radiologists from the Department of Radiology, Frederiksberg Hospital, will read all scans, and the physician from The Parker Institute Department of Rheumatology will inform the participants of the MRI findings by telephone or by an informative letter mailed to the patients. The final MRI report will also be sent to the participant’s GP. The participants may be excluded if necessary according to the exclusion criteria after the MRI. Omission on the request of the participant of the follow-up scans will not have an effect on participation in the study.

### Intervention

**Figure 2:**
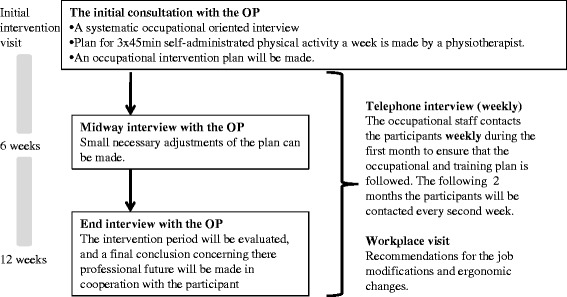
The 12 weeks occupational medicine intervention.

All participants will receive conventional medical care according to the regional guidelines and best practices. This consists of a brief instruction in exercises and pharmacologic management with the use of analgesics and anti-inflammatory agents and/or contact with a physiotherapist. The study’s additional occupational medicine intervention will last 3 months and include: 1) an initial consultation with the OP; 2) a workplace visit, if required; 3) a consultation with a physiotherapist; 4) a weekly telephone interview the first month and every second week the following two months with focus on adherence to the intervention plan; 5) midway interview with the OP with focus on return to/retention at work; 6) after three months a session with the OP evaluating the workplace-oriented intervention and the physical activity and concluding with further guidance. The intervention is illustrated in Figure 2.

#### The initial consultation with the OP

The aim of this consultation is to establish knowledge about the participant’s work situation and uncover opportunities to remove barriers for maintaining the participant in his job. A systematic interview will be performed and will comprise the following: 1) a detailed description of the participant’s daily work, including work function, work tasks, general work demand, work tasks that aggravate the back pain and organisational problems. 2) If there is a potential need for support for social problems such as job insecurity, advice will be given and registered. 3) Advice to the participant regarding actual level of physical activity will be given. The consultation takes approximately one hour and will result in an agreed intervention plan.

The intervention plan can include: 1) workplace visit; 2) advice concerning possibilities for more variation during the work day; 3) temporary or permanent ergonomic changes; 4) change of work tasks and job routines; 5) need for job training; 6) transfer to other job tasks; 7) rehabilitation or additional formal education; 8) a schedule for gradual return to work; 9) advice on avoiding work loads that aggravate the low back problems. If necessary this plan will be adjusted at the following telephone interviews. A short communication form is sent to the GP to prevent conflicting advice to the participant in the return to/retain at work process and, if necessary, the social authorities and employment exchange will be contacted.

#### Workplace visit

The workplace visit will be arranged in collaboration with the participant, the supervisor at the workplace and the occupational staff. Recommendations will be based on the active participation and strong commitment of both the participants and supervisor at the workplace. The aim of the workplace visit is to provide information for recommendations such as job rotations, the need for ergonomic initiatives, modifying the work, such as temporary or permanent exemption from special tasks and personal assistance. The recommendations for the job modifications and ergonomic changes are presented for the participants and the supervisor at the workplace and are reported in a structured scheme. The participant and the supervisor are responsible for implementing the plan.

#### Consultation with a physiotherapist

In addition to the occupational medicine plan, three 45-minute self-administered physical activity sessions a week are planned by a physiotherapist. The goal for these physical activities is that they should be easily integrated into the participant’s life style (for example, sports activities with the kids, swimming, fast walking, running, bicycling and the like).

#### Telephone interview

The participants are contacted by the physiotherapist weekly during the first month to ensure that the occupational and training plans are followed; if necessary, adjustments of the plan will be made. For the following two months the participants are contacted every 14 days.

#### Six weeks midway interview with the occupational staff

This interview will focus on the potential of the participants to stay at work; small necessary adjustments of the plan can also be made. This interview will last about 45 minutes.

#### Three months consultation with the OP

At this consultation the intervention period will be evaluated, and in cooperation with the participant a final conclusion concerning her professional future will be made. Participants who declare that they have not followed the intervention plan will be defined as non-adherent to the intervention. This consultation will last approximately one hour.

All intervention participants are encouraged to continue receiving healthcare for any non-back pain-related conditions from their usual primary care providers during the intervention.

### Primary outcome

The primary outcome of this study is retention at work after occupational medicine intervention, measured as the accumulated duration of self-assessed sick leave due to LBP over three and six months. The sick leave data is assessed weekly on a paper-based sick leave diary. The participants have to answer the following question: *How many days have you been on sick leave due to LBP during the past week?* For the first six months, all participants will weekly receive a text message with a reminder to answer the sick leave question in the diary. Additional participants can be contacted by telephone every second week, and the accumulated sick leave will be registered by an independent observer.

### Secondary outcomes

Secondary outcomes will focus on sick leave and different aspects associated with LBP with the potential to affect the ability to return to/stay at work. Sick leave is additionally assessed by using data from the Danish National Register on Public Transfer Payments (Danish acronym DREAM). The DREAM register includes week-by-week records of any type of public transfer payment at an individual level. Sick leave periods of less than two consecutive weeks are not recorded in the DREAM register. Pain will be evaluated by using the 13-item painDETECT questionnaire, which includes measurements of LBP on an ordinal 11-point numerical rating scale (NRS: 0 = no LBP; 10 = worst LBP possible) [26]. The validated 24-item Roland Morris Disability Questionnaire will measure participant-rated LBP disability [27]. The participant’s work ability will be rated by three selected questions from the seven-item Work Ability Index questionnaire [28] and by an occupational standard baseline questionnaire collecting information on demographics, education level, leisure-time physical activity and the psychosocial work environment. General health status is collected with the Short Form Health Survey (SF-36) to assess changes in physical functional (SF-36 physical function subscale) and mental health (SF-36 mental health subscale) [29,30]. The psychosocial aspect of LBP will be measured with the validated Fear Avoidance Beliefs Questionnaire, a 16-item instrument measuring back-pain fears on two subscales related to physical activity and work which are rated from 0 (completely disagree) to 6 (completely agree) [31] [http://fysio.dk/fafo/Nyheder/Fear-Avoidance-Beliefs-Questionnaire]. Overall satisfaction with the intervention for the participants of this group is rated at each follow-up visit on an 11-point NRS with the anchors ‘not at all satisfied’ to ‘extremely satisfied’. The baseline MRI evaluation represents a descriptive outcome, and the lumbar degeneration is measured as a sum score of the discus at all lumbar levels [32,33].

If a participant is unable to attend the half-year visit in person, the primary outcome measures and a subset of secondary outcomes can be administered via paper-based questionnaires sent to the participant’s home with a stamped return envelope. All primary and secondary outcome assessments are also presented in Table 1.

**Table 1:** **Protocol schedule of forms and procedures**

	**Baseline**	**6 months primary outcome**	**1 year**	**2 year**
The occupational medicine standard baseline questionnaires	X			X*
Job category ¶	X	X	X	X
Physically work load in current physically demanding job, Grading (1-4) ¶	X	X	X	X
Sick leave due to LBP the last year, days	X††	X†	X††	X††
Clinical examination	X	X	X	
Pain, VAS: (0-100 mm)	X	X	X	X
SF-36, score (0-100)	X	X	X	X
Work Ability Index Questionnaire 3 items	X	X	X	X
Fear Avoidance Beliefs, Work Subscale (0-42)	X	X	X	X
Fear Avoidance Beliefs, Physical Activity subscale (0-24)	X	X	X	X
Pain categorization, PainDETECT®, score (0-38)	X	X	X	X
Roland Morris Disability, score (0-24)	X	X	X	X
Magnetic resonance imaging, semi-quantitative scores	X		X	
Satisfaction with the intervention, NRS (1-10)		X	X	X

*Selected questions. †Sick leave due to LBP measured weekly during the first six months previously specified in the ’primary outcome‘ section. ††Patient-reported outcome. ¶ Additional reported at the telephone interview.

### Sample size

For a two-sample pooled *t*-test of a normal mean difference with a two-sided significance level of 0.05 (*P* < 0.05), assuming a common standard deviation of 17 days [17,34], a sample size of 127 participants per group is required to obtain a power of at least 80% to detect a mean difference of 6 days. Expecting some drop-outs during the trial period (less than 20%), it was decided for pragmatic reasons to enrol 300 participants in total (that is, 150 participants in each group). Including 300 participants in the intention-to-treat population, based on the assumptions above, corresponds to a statistical power of 86.1% to detect a mean difference of 6 days.

### Randomisation

The allocation occurs through a 1:1 ratio by a predetermined restricted randomisation scheme. The participants are stratified according to their age at enrolment (<40 years or ≥ 40 years) and gender (male or female). The eligible participants are randomly assigned in permuted blocks of 4 and 6, according to computer-generated random numbers, to be enrolled in either the intervention or control group. The randomisation is administered by predetermined sequentially numbered, opaque envelopes stored in a locked cabinet in a secure location. The randomisation is administered by Good Clinical Practice (GCP)-trained personnel not involved in the outcome measurement or the intervention.

### Blinding

The physicians performing the clinical examinations of the participants as part of the assessment of this trial are not taking part in occupational medicine intervention [23]. In addition, we will also try to make the physicians of assessment blinded for the allocation by instructing the participants not to mention their allocation in any follow-up visits. The OPs will, as coordinators of the study, not be blinded to the participant’s allocation but to the participant’s outcome data. The primary outcome is self-administered by a paper-based diary, and most of the secondary outcome measures are self-administered questionnaires on touch screen without involvement of the observers. In case of need of a telephone interview, this will be performed by personnel not connected to the intervention.

### Data handling

The Parker Institute’s statistical unit handles all data-management procedures and the trial central databases (SQL Server 2008 Express; Microsoft). The user-friendly touch screen interface obtains all self-reported outcome questionnaire measures from which all data are transferred directly to the study database [25]. During the intervention phase only the database manager from the statistical unit at the Department of Rheumatology has access to the database. Paper-based forms will only be used in case of server breakdown and the subsequent data entry will be performed by personnel not involved in the intervention. Research records and data collection are obtained according to the Danish Personal Data Act (DPA) guidelines and recommendations given in their study approval (DPA approval number 2014-41-2673).

The local ethical committee and an external advisory board at the Parker Institute will ensure patient safety and the highest possible data quality. According to Danish law, independent physicians from the local ethical committee and/or the National Health and Medicine Authorities can conduct monitoring visits on a regular basis.

### Statistics

Data analyses will be performed using the SAS System for Windows (Release 9.3, SAS Institute Inc., Cary, NC, USA). Descriptive statistics of participant baseline characteristics will be presented for both groups to assess their comparability. Continuous data will be analysed using an Analysis of Covariance (ANCOVA), with a factor for Group, using the baseline value as a covariate to reduce the random variation and increase the statistical power. Unless stated otherwise, results will be expressed as the difference between the group means with 95% confidence intervals (CIs) based on a General Linear Model (GLM) procedure. *P*-values will only be reported if appropriate. We will attempt to follow up all randomised participants, even if they withdraw from allocated treatment. The main analyses will be on sick leave after 6 months where participants will be analysed according to the allocated group. The Cox proportional hazard model will be used to analyse differences in time until return to work, including the intervention group as the only independent variable.

The cost-effectiveness analysis of our occupational medicine intervention will include predictors of return to work, based on questionnaire outcomes, the MRI evaluation and clinical examination. This study is testing an additional intervention to the treatment provided by the participants’ GP; therefore, the direct intervention costs included the costs of the workplace and our occupational medicine intervention. This will be compared to the costs of production losses estimated by a price of production loss of a worker per day. Further direct non-health care costs will be included (such as pension expenses, loss of productivity, schooling and redeployment).

### Ethics

The trial will be conducted in accordance with the human rights and dignity of the participants as reflected in the Declaration of Helsinki. Individuals can only participate in the study after signing the informed consent disclosure. All participants are insured according to the national health insurance and are informed about this before enrolment. Participants receive no financial compensation for participating in this study. No published material will contain patient identifiable information.

### Anticipated risks and benefits

All participants will receive usual standard care, and no treatment will be withheld to participants in this trial. Both allocation groups receive active treatments with previously demonstrated efficacy and no known iatrogenic effects. This trial may benefit individual participants, since occupational medicine intervention is not generally available for the majority of people with LBP. Participants will receive an intensive level of monitoring so that if any participants experience deterioration of their LBP it will be identified and they will be referred to appropriate care in collaboration with their GP. To date there are no reported harms or adverse effects related to MRI if the procedure follows the international and national guidelines [35]. The standing position G-scan may be associated with dizziness and eventually orthostatic syncope, due to the standing posture. This complication will be met by an external pneumatic compression system applied to the participant’s legs which substantially reduces these events [36]. Research clinicians, physiotherapists and other staff are instructed to report all potential adverse effects to the project coordinator (AIK or BBH).

## Discussion

There are many guidelines available on the management of LBP, but they provide limited guidance on the occupational aspects. This study protocol describes a randomised controlled trial designed to evaluate the effectiveness and feasibility of an occupational medicine intervention for retaining at work workers in physically demanding jobs with LBP. If the results are positive and show cost-effectiveness, the project can lead to a future change in policy for individuals with LBP and physically demanding jobs with increased collaboration between workers, workplaces and health professionals.

## Trial status

Participant recruitment began March 2014 and is ongoing at the time of this manuscript’s submission.

## Abbreviations

DPA: Danish Data Protection Act
FABQ: Fear Avoidance Beliefs Questionnaire
GCP: Good Clinical Practise
GoBack trial: retention in physically demanding jobs with low back pain
GP: general practitioner (family medicine physician)
ICD-10: International Classification of Diseases 10th revision
LBP: low back pain
MRI: magnetic resonance imaging
NRS: numerical rating scale
OP: occupational physician
OPUS: Danish patient record system
PACS: picture archiving and communication system
RMDQ: Roland-Morris Disability Questionnaire
SF-36: 36-item short-form health-survey questionnaire
TSE-T1: T1-weighted turbo spin echo
TSE-T2: T2-weighted turbo spin echo
VAS: visual analogue scale
